# Outcomes of Double Balloon-Enteroscopy in Elderly vs. Adult Patients: A Retrospective 16-Year Single-Centre Study

**DOI:** 10.3390/diagnostics13061112

**Published:** 2023-03-15

**Authors:** Margherita Trebbi, Cesare Casadei, Silvia Dari, Andrea Buzzi, Mario Luciano Brancaccio, Valentina Feletti, Alessandro Mussetto

**Affiliations:** Gastroenterology Unit, Santa Maria delle Croci Hospital, 48121 Ravenna, Italy

**Keywords:** capsule endoscopy, double-balloon enteroscopy, elderly, enteroscopy, small bowel endoscopy

## Abstract

**Background and Aim**: Double-balloon enteroscopy (DBE) is a well-established procedure for direct visualisation of the entire small bowel mucosa, and, in contrast with other imaging techniques, allows to perform biopsies and therapeutic interventions. The aim of this study was to evaluate the indications, diagnostic yield, therapeutic yield, and complications of DBE in a cohort of consecutive patients according to patients’ age. **Methods**: We conducted a retrospective study of consecutive patients who underwent DBE in our endoscopy unit between January 2006 and December 2021. **Results**: A total of 387 consecutive patients who underwent 460 DBE procedures were included. Mean age of the patients was 63 years. The overall diagnostic yield was 67.6%; vascular lesions were the predominant endoscopic findings (31.5%), followed by polyps or neoplastic masses (17.6%). Older patients (≥65 years) showed statistically higher rates of clinically relevant findings than adult patients (18–65 years) (*p* = 0.001). Crohn’s disease and polyps or neoplastic masses were more frequent in the younger group (*p* = 0.009 and *p* = 0.066, respectively), while vascular lesions and non-specific inflammation were the most common findings in the older group (*p* < 0.001 and *p* < 0.001, respectively). The therapeutic intervention rate was 31.7%. Rates of endoscopic treatment were significantly higher in the older group (*p* < 0.001). Total complications occurred in five procedures (1.1%). **Conclusion**: In clinical practice, DBE is an efficient diagnostic and therapeutic tool with a high safety profile, particularly in the elderly population.

## 1. Introduction

Direct evaluation of the small bowel has been a great challenge for decades, mainly caused by its anatomical structure, location, and length.

Two main techniques made the small bowel’s exploration possible: video capsule endoscopy (VCE) and device-assisted enteroscopy (DAE), both of which are constantly developing [1,2,3,4].

In 2000, VCE was introduced as a non-invasive manner for direct visualization of the small bowel, with high safety and a high rate of complete checking of the small intestine; however, its greater limitation is that it is a purely diagnostic procedure. 

Device-assisted enteroscopy (DAE) is a generic term that includes any endoscopic technique for the exploration of the small bowel with a system of assisted progression (a balloon, overtube, or other stiffening device). DAE includes double-balloon enteroscopy (DBE, Fujinon Inc., Saitama, Japan), single-balloon enteroscopy (SBE, Olympus Optical Co., Tokyo, Japan), spiral enteroscopy (PowerSpiral; Olympus Medical, Tokyo, Japan), and balloon-guided endoscopy (NaviAid AB device, SMART Medical Systems Ltd., Ra’anana, Israel) [1,2].

As compared to VCE, DAE is an endoscopic method that enables not only a direct visualization of the small bowel, but also biopsy sampling and therapeutic interventions such as mucosectomy or haemostasis [3]. 

A complete visualization of the small bowel can be achieved with a combined approach by oral and anal route. During enteroscopy, a clip or a tattoo can mark the most distal point reached to establish whether “panenteroscopy” has been achieved. DAE can detect lesions missed by VCE, so that with the combination of these two techniques, the positive findings rate could reach 92.5% in some studies [5]. 

The main application of DAE is small bowel bleeding. In this case, the procedure is usually preceded by a diagnostic VCE or imaging study that detects the source of bleeding; however, DAE is indicated also in patients with obscure gastrointestinal bleeding, whether occult or overt. Another important application field of enteroscopy is Crohn’s disease (CD): recent guidelines by the European Society of Gastrointestinal Endoscopy (ESGE) recommends its use for diagnosis in patients with suspected small bowel localization of CD with non-contributory ileocolonoscopy, but also for therapeutic intervention, such as dilation of strictures or retrieval of retained capsules [2]. Furthermore, DAE allows endoscopic retrograde cholangiopancreatography (ERCP) for treatment of pancreaticobiliary diseases in patients with surgically altered anatomy, such as Roux-en-Y entero-enteric anastomosis [2,6]. Other indications of DAE include small bowel tumours or polyposis (detection, sampling, and tattooing of neoplastic lesions, polypectomy of adenomatous lesions, or hamartomas in Peutz–Jeghers syndrome), non-responsive or refractory celiac disease, retrieval of foreign bodies, or percutaneous endoscopic jejunostomy [2]. The main limits of DAE are that it could be time consuming, it is an invasive procedure, and a learning curve is needed. 

Double-balloon enteroscopy (DBE) was first described by Yamamoto et al. in 2001 [7].

DBE is a system consisting of an endoscope and an overtube, each of which is provided with a latex balloon on the tip that can be inflated and deflated using a pump. The endoscope is inserted further along the small intestine by inflating and deflating the two balloons and using push-and-pull manoeuvres. In comparison to the other DAE systems, DBE showed a deeper insertion rate compared with manual spiral enteroscopy [8] and a higher total enteroscopy rate when compared with SBE [9].

In the last decade, various studies on DBE have reported the indications, diagnostic and therapeutic yield, and complications rate for small bowel diseases worldwide, in particular in the setting of bleeding [3,5,10,11,12,13,14,15,16,17,18,19,20,21]. Double-balloon enteroscopy can be performed with acceptable safety in the elderly, even with high ASA class [22], but few retrospective studies had focused on the above-mentioned outcomes of DBE in relation to different ages, mostly from China [5,10,11,23,24,25,26,27].

In our study, we retrospectively analyzed the indications, endoscopic findings, therapeutic interventions, and complications among patients who underwent DBE over 16 years in our hospital, with particular regard to different age groups. 

## 2. Materials and Methods

### 2.1. Data Collection and Study Design

We conducted a retrospective review of all consecutive patients who underwent DBE at the Santa Maria delle Croci Hospital of Ravenna, between 1 January 2006 and 31 December 2021. We excluded patients aged < 18 years. 

All features including demographic data, indications and previous diagnostic examinations, sedation type, route of insertion, insertion length, endoscopic findings, applied interventions, and complications related to the procedure were noted.

All the procedures were performed by two experienced endoscopists, under fluoroscopic guidance, using the therapeutic Fujinon Double-Balloon Enteroscopy System (Fujinon Inc., Saitama, Japan) with a working length of 200 cm, a soft overtube with a length of 140 cm, and a 2.8 mm working channel. A push-and-pull technique was adopted. Both CO2-insufflated and water aided techniques were used, depending on endoscopist’s preference. 

Sedation was applied by an anaesthesiologist using conscious sedation (benzodiazepine–opiate combination), deep sedation with propofol, or general anaesthesia with endotracheal intubation. 

The insertion route was determined based on the estimated location of the target lesion or the bleeding site according to the previous studies’ guidance or clinical symptoms.

Oral DBE was performed with 12 h fasting, while for anal DBE, the patient underwent a standard colonic lavage with a 2–4 L of polyethylene glycol–electrolyte solution on the day before the endoscopic procedure [28]. 

The insertion length was estimated by the sum of every insertion session as first described by May et al. [29], as European Society of Gastrointestinal Endoscopy recommends in its specific technical review [28]. The small bowel segment reached was subsequently estimated according to both the depth of insertion and the previous studies’ guidance. Reasons for impossibility to advance further the enteroscope in the bowel were noted. 

Obscure gastrointestinal bleeding (OGIB), either overt or occult, was defined as patients not found to have a source of bleeding after performance of standard upper and lower endoscopic examinations, small bowel evaluation with VCE, or radiographic testing [30].

The target lesions were defined as compatible with previous findings of other studies (imaging or VCE), tumour-like lesions, active bleeders, or active inflammatory lesions. 

Indian ink was used for tattooing the furthest point of insertion without reaching the target lesion or when a neoplastic lesion was found. 

Any DBE-associated complications were referred as event-associated DBE procedures, and severity was graded according to ASGE lexicon. 

Patients were finally divided into two groups by their age, according to the definition of elderly by the WHO [31], in adult (age 18–65 years) and elderly group (age ≥ 65 years). 

### 2.2. Statistical Analysis

All statistical analyses were performed using IBM SPSS Statistics version 26.0. Continuous variables were expressed as mean ± standard deviation (SD) and categorical variables were expressed as frequency or percentages. Categorical variables were analyzed by the χ^2^ or Fisher’s exact test, while continuous variables were analyzed by the Student’s t-test. A *p* < 0.05 was considered statistically significant. Positive diagnostic yield was defined as a significant endoscopic finding on DBE that was considered to be clinically relevant to the indication for endoscopy. Therapeutic yield was defined as any endoscopic intervention, apart from biopsies. 

## 3. Results

The study sample included 460 procedures in 387 consecutive patients. The mean age of the patients (female 45.2%) was 63 (min 18, max 93). Of the 460 procedures, the access route was anterograde in 349 (75.9%) patients and retrograde in 111 (24.1%). 

The mean length of extension in the anterograde procedure was 222 cm (SD 98 cm, min 30 cm–max 580 cm) and 88 cm with the retrograde approach (SD 48 cm, min 10 cm–max 250 cm). 

The main indication for DBE procedures was a positive VCE examination (67.8%), followed by a positive radiologic study (16.3%). Other indications were OGIB (7.8%), Crohn’s disease (3.9%), abdominal pain (2.4%), and refractory iron deficiency anaemia (1.7%). Of the patients that presented a positive video capsule endoscopy, 27.8% had small bowel angioectasias (27.8%), 19,3% had polyps or neoplastic lesions, 11.1% presented with blood in small bowel lumen without any clear pathological lesion, and 9.6% had mucosal inflammation. 

**Figure 1:**
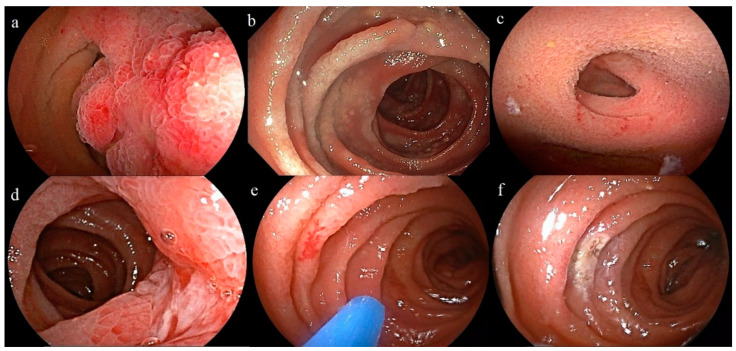
Examples of pathological findings. (**a**) Jejunal hamartoma. (**b**) Jejunal lymphoma. (**c**) Meckel’s diverticulum. (**d**) Jejunal Crohn’s disease. (**e**) Jejunal angioectasia (**f**) Jejunal angioectasia after treatment with argon plasma coagulation.

A total of 149 (32.4%) DBE were normal, while in 311 (67.6%) cases, a clinically relevant finding (69.6% for the oral route and 61.3% for the anal route) was detected; the most frequent pathological findings were vascular lesions (31.5%), in particular angioectasias (26.5%) and polyps or neoplastic masses (17.6%). Other frequent DBE findings were non-specific inflammation (11.7%) and Crohn’s disease (5%). Some typical pathological findings are shown in Figure 1. 

Endoscopic treatment was performed in 146 (7%) patients in which haemostasis (27.4%) was the prevalent intervention, mainly through argon plasma coagulation (112 cases) and less frequently with adrenaline and clips (14 cases). Endoscopic mucosal resection (EMR) was carried out in 20 (4.3%) cases. 

Endoscopic biopsies were taken in 139 cases (30.2%) and tattooing of the distal site of intubation was performed in 142 procedures (30.9%). 

The overall complication rate was 1.1% (5/460), with a severe complication rate of 0.7% (3/460). Fever during the day after the procedure was observed in two patients. three patients had severe complications, all of them during anterograde DBE: one perforation, one acute myocardial infarction, and one aspiration pneumonia. No case of acute pancreatitis nor mortality were observed. The data are listed in Table 1.

### DBE in Different Age Groups

We performed a retrospective analysis of the data according to age of patients, divided in two groups: adults (<65 years, 209/460, 45.4%) and elderly (≥65 years, 251/460, 54.6%). Data are shown in Table 2.

In the older group, more anterograde procedures were performed (*p* = 0.011) and more DBE were preceded by VCE study (*p* = 0.001). DBE was more often guided by radiology findings in the younger group (*p* = 0.045) and by VCE findings in the older group (*p* = 0.003). In particular, DBE indications such as inflammation, polyps, or masses at VCE were more common in the younger group (*p* = 0.026 and *p* = 0.006, respectively), while vascular lesions such as angioectasias were more frequent in the older group (*p* < 0.001). 

**Figure 2:**
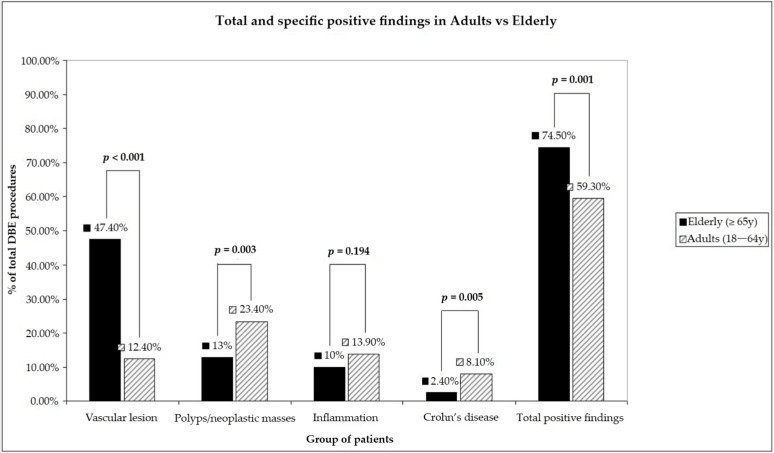
Difference in term of diagnostic yield between elderly and adults.

The overall diagnostic yield of DBE was significantly different between the two groups (*p* = 0.001, Figure 2): 59.3% in the younger group and 74.5% in the older group. The detection rates for Crohn’s disease and for polyps or other small bowel masses were significantly higher in the younger group (*p* = 0.005 and *p* = 0.003, respectively). Instead, vascular lesions were more frequently detected in the older group (*p* < 0.001). Although the detection of aspecific inflammation was more frequent in the younger group (13.9% vs. 10%), there was no statistically significant difference between the two groups (*p* = 0.194). Biopsies and tattooing were more often performed in the younger group (*p* < 0.001 and *p* = 0.034, respectively). 

**Figure 3:**
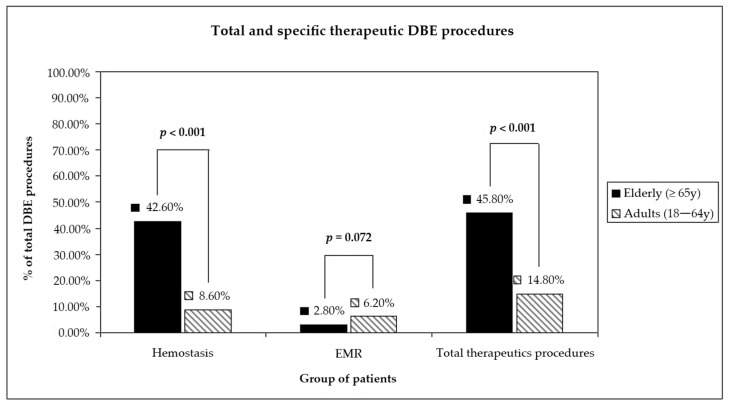
Difference in term of therapeutic yield between elderly and adults.

The therapeutic yield was significantly different between the two groups, too (*p* < 0.001) (Figure 3): 14.8% in the younger group and 45.8% in the older group. Endoscopic haemostasis was performed more often in the older group (*p* < 0.001), while rates of endoscopic polypectomy were not significantly different (*p* = 0.072), even though EMR was mostly performed in the younger group (13 cases vs. 7 cases in the older group). No statistically significant difference was found among complications between the two groups (*p* = 0.664). 

## 4. Discussion

Double-balloon enteroscopy is a well-established procedure, born more than twenty years ago, that enables direct visualization of the small bowel, and moreover overcomes the limitation of capsule endoscopy, since it allows endoscopic treatment of small bowel lesions, including haemostasis and resection of polyps. To our knowledge, this is one of the largest European single centre retrospective studies reporting DBE indications, results, and complications in a cohort of consecutive patients from 2006 to 2021.

The diagnostic yield of DBE shows a great variable range in previous published studies that varies between 43% and 81%. According to a systematic review by Xin et al., of 66 published studies, the pooled detection rate of DBE of all small bowel disease was 68.1%. In particular, the rate in Europe was about 66.7% [14], which is in line with our study.

In our report, the anterograde route showed a more accessible and incisive approach than the retrograde one: the diagnostic yield of anterograde DBE reached 69.6%, while the detection rate of the retrograde approach was lower (61.3%), which is consistent with another previous retrospective study by Shelnut et al. (62%) but greater than that reported in the literature (from 39 to 47%) [15].

In Western countries, vascular lesions account for nearly two-thirds (65.9%) of positive findings, followed by inflammatory lesions (15.7%) and neoplastic lesions (14.4%) [14]. In our series, vascular lesions, mainly angioectasias, were also the most frequent findings (46.6%), followed by polyps or neoplastic lesions (26%) and aspecific mucosal inflammation (17.4%). Active Crohn’s disease was found in 7.4% of DBE, which is in line with Western countries and lesser than Asian reports [1,2,5].

Similar to the diagnostic yield, the average pooled therapeutic rate in the published literature is variable from 17% up to 42% [5,15,17,18]. In our study, the therapeutic yield was 31.7% (146/460). The main therapeutic interventions were endoscopic haemostasis, in the majority of cases with APC and less frequently with adrenaline and clips, likewise in the previous European literature [18,19]. In 20 cases (4.3%), a snare polypectomy was performed. 

Currently, people worldwide may reach older ages because of advances in medical sciences. According to the latest demographic data released in 2023 [32], the elderly represents 23.8% of Italy’s population, and this percentage is increasing every year, representing one of the oldest populations worldwide. There are few studies that analyse DBE performance by age groups, mostly carried out in Asian countries [5,10,11,23,27]. A systematic review and meta-analysis by Chetcuti Zammit et al. on DAE demonstrated a higher diagnostic and therapeutic yield in the elderly compared to adult patients [24]. This finding has recently been corroborated by an Italian monocentric retrospective study, in the specific setting of small bowel bleeding [25]. 

Considering the whole spectrum of small bowel diseases, our report showed a difference in the diagnostic yield between age groups (*p* = 0.037); in particular, in the older group, vascular lesions and inflammation appeared to be statistically more frequent (*p* < 0.001 and *p* < 0.001, respectively). Meanwhile, the younger group showed a higher rate of Crohn’s disease (*p* = 0.009) and polyps or neoplastic masses, although the latter did not reach a significant difference (*p* = 0.066). Except for one case series by Johnston and colleagues that reported a higher rate of malignancy in younger patients (cut off ≤ 55 years) [33], most studies did not show a significant difference in the incidence of small bowel tumours according to the age of the patients [23,34,35]. In our report, we found a higher rate of masses in the younger group. This is mainly due to the fact that neoplastic masses were grouped together with polyps in general, which include hamartomas in patients with Peutz–Jeghers syndrome, juvenile polyps, and adenomatous polyps. Moreover, there is a relevant variance in the definition of younger versus older age groups between studies that could condition results.

Probably because of the above-mentioned findings, biopsies were taken most frequently in the adult group (*p* = 0.001). 

Furthermore, the therapeutic yield was found to be significantly higher in the older group (*p* < 0.001), especially regarding endoscopic haemostasis. This is probably related to the higher frequency in this group of angioectasias and other haemorrhagic sources as indications (*p* < 0.001 and *p* = 0.01, respectively) and, accordingly, of vascular lesions diagnosed during the procedure. 

Major complications related to DBE are uncommon, ranging from 0% to 1.2% based on the previous literature [3,5,14,16,17,18,19,20,21,27,36], with a minor complication rate that can reach 9.1% [14]. According to our study, major complications were observed to be rare (3/460, 0.7%), so that DBE procedure can be defined as a safe intervention. When comparing with a younger population, elderly patients usually have more comorbidities and higher use of anticoagulation and antiplatelet agents. In addition, geriatric patients have a greater risk of oversedation and aspiration. A recent review found that the highest complication rated reported in the elderly was up to 5.4% [37]. Remarkably, no difference was found between the two age groups in regard to the complication rate: all minor complications occurred in the younger group, and among major complications, one acute myocardial infarction occurred in the younger group, while one case of aspiration pneumonia and one perforation were reported in the older group. 

Our study had some limitations: firstly, the retrospective design, and secondly, although there were large sample sizes of patients, this was only a single referral centre, thus limiting the generalizability of findings to smaller community settings. Moreover, a few patients had undergone initial imaging procedures on other centres and then referred to our unit, sometimes after more than four weeks, and this could alter the diagnostic and therapeutic rates in these patients, especially in regard to the detection and treatment of vascular lesions. 

Notwithstanding, our study comprises a relatively large clinical sample of patients undergoing DBE (*n* = 460) and this is one of the few European single centre retrospective analyses that specifically analyses performance of DBE in the elderly population as compared to adult patients. 

In summary, our study reinforces the diagnostic and therapeutic utility of DBE in a large cohort of patients with suspected small bowel diseases, especially in the setting of the elderly population, where DBE shows high efficacy and safety.

## Figures and Tables

**Table 1:** Characteristics, diagnostic yield, therapeutic yield and complications of double-balloon-assisted enteroscopy.

Enteroscopy Characteristics	N	%
**Route**		
Anterograde	349	75.9%
Mean length in cm	222 cm	
Retrograde	111	24.1%
Mean length in cm	88 cm	
**Endoscopic findings**		
Normal	149	32.4%
Pathologic	311	67.6%
Vascular lesion	145	31.5%
Polyp or tumour mass	81	17.6%
Inflammation	54	11.7%
Crohn’s disease	23	5.0%
Others *	8	1.7%
**Biopsies taken**	139	30.2%
**Tattooing**	142	30.9%
**Intervention**	146	31.7%
Hemostasis	126	27.4%
Polypectomy	20	4.3%
**Complications**	5	1.1%
Fever	2	0.4%
Acute myocardial infarction	1	0.2%
Ab ingestis pneumonia	1	0.2%
Perforation	1	0.2%

* stricture/stenosis, prominent lymphatics, lymphoma, retained capsule, external compression, Meckel diverticulum.

**Table 2:** Comparison between adult and elderly patients: characteristics, indications, diagnostic and therapeutic yield, and adverse events.

	AGE < 65 Years (209/460, 45.4%)	AGE ≥ 65 Years (251/460, 54.6%)	*p* Value
**Sex**			
Male	114	142	0.663
Female	95	109	
**Route**			
Anterograde	147	202	**0.011**
Retrograde	62	49	
**CE**	150	212	**0.001**
**Indications**			
Radiology	42	33	**0.045**
CE	127	185	**0.003**
Inflammation	27	17	**0.026**
Polyp/Tumour	52	37	**0.006**
Angioectasia	32	96	**<0.001**
Blood in lumen (without a clear lesion)	16	35	**0.032**
Symptoms	8	3	0.122
Crohn’s disease	13	5	**<0.001**
OGIB	15	21	0.636
Iron-deficiency anaemia	4	4	1.000
**Findings**	124 (59.3%)	187 (74.5%)	**0.001**
Vascular lesions	26	119	**<0.001**
Inflammation	29	25	0.194
Polyp/Tumour	49	32	**0.003**
Crohn’s disease	17	6	**0.005**
Other	3	5	0.733
**Biopsy**	82	57	**<0.001**
**Tattoo**	75	67	**0.034**
**Therapeutic**	31	115	**<0.001**
APC/clip	18	107	**<0.001**
EMR	13	7	0.072
**Complications**	3	2	0.664

## References

[1] Pennazio M., Spada C., Eliakim R., Keuchel M., May A., Mulder C.J., Rondonotti E., Adler S.N., Albert J., Baltes P. (2015). Small-bowel capsule endoscopy and device-assisted enteroscopy for diagnosis and treatment of small-bowel disorders: European Society of Gastrointestinal Endoscopy (ESGE) Clinical Guideline. Endoscopy.

[2] Pennazio M., Rondonotti E., Despott E.J., Dray X., Keuchel M., Moreels T., Sanders D.S., Spada C., Carretero C., Valdivia P.C. (2023). Small-bowel capsule endoscopy and device-assisted enteroscopy for diagnosis and treatment of small-bowel disorders: European Society of Gastrointestinal Endoscopy (ESGE) Guideline—Update 2022. Endoscopy.

[3] Saygili F., Saygili S.M., Oztas E. (2015). Examining the whole bowel, double balloon enteroscopy: Indications, diagnostic yield and complications. World J. Gastrointest. Endosc..

[4] Alemanni L.V., Fabbri S., Rondonotti E., Mussetto A. (2022). Recent developments in small bowel endoscopy: The “black box” is now open!. Clin. Endosc..

[5] Wang P., Wang Y., Dong Y., Guo J., Fu H., Li Z., Du Y. (2021). Outcomes and safety of double-balloon enteroscopy in small bowel diseases: A single-center experience of 1531 procedures. Surg. Endosc..

[6] Skinner M., Popa D., Neumann H., Wilcox C.M., Mönkemüller K. (2014). ERCP with the overtube-assisted enteroscopy technique: A systematic review. Endoscopy.

[7] Yamamoto H., Sekine Y., Sato Y., Higashizawa T., Miyata T., Iino S., Ido K., Sugano K. (2001). Total enteroscopy with a nonsurgical steerable double-balloon method. Gastrointest. Endosc..

[8] Despott E.J., Murino A., Bourikas L., Nakamura M., Ramachandra V., Fraser C. (2015). A prospective comparison of performance during back-to-back, anterograde manual spiral enteroscopy and double balloon enteroscopy. Dig. Liver Dis..

[9] Lipka S., Rabbanifard R., Kumar A., Brady P. (2015). Single vs. double balloon enteroscopy for small bowel diagnostics: A systematic review and meta-analysis. J. Clin. Gastroenterol..

[10] Chen W.G., Shan G.D., Zhang H., Yang M., Lin L., Yue M., Chen G.W., Gu Q., Zhu H.T., Xu G.Q. (2016). Double-balloon enteroscopy in small bowel diseases: Eight years single-center experience in China. Medicine.

[11] Lu L., Yang C., He T., Bai X., Fan M., Yin Y., Wan P., Tang H. (2022). Single-centre empirical analysis of double-balloon enteroscopy in the diagnosis and treatment of small bowel diseases: A retrospective study of 466 cases. Surg. Endosc..

[12] Wang Y.X., Bian J., Zhu H.Y., Dong Y.H., Fang A.Q., Li Z.S., Du Y.Q. (2019). The role of double-balloon enteroscopy in reducing the maximum size of polyps in patients with Peutz-Jeghers syndrome: 12-year experience. J. Dig. Dis..

[13] Hedge S.R., Iffrig K., Li T., Downey S., Heller S.J., Tokar J.L., Haluszka O. (2010). Double-balloon enteroscopy in the elderly: Safety, findings and diagnostic and therapeutic success. Gastrointest. Endosc..

[14] Xin L., Liao Z., Jiang Y.P., Li Z.S. (2011). Indications, detectability, positive findings, total enteroscopy, and complications of diagnostic double-balloon endoscopy: A systematic review of data over the first decade of use. Gastrointest. Endosc..

[15] Shelnut D.J., Sims O.T., Zaibaq J.N., Oh H., Venkata K.V., Peter S. (2018). Predictors for outcomes and readmission rates following double balloon enteroscopy: A tertiary care experience. Endosc. Int. Open.

[16] Hong S.N., Kim E.R., Ye B.D., Jang H.J., Jeon S.R., Park S.J., Im J.P., Kim J.H., Choi C.H., Choi H. (2016). Indications, diagnostic yield, and complication rate of balloon-assisted enteroscopy during the first decade of its use in Korea. Dig. Endosc..

[17] Dişibeyaz S., Suna N., Kuzu U.B., Saygılı F., Öztaş E., Ödemiş B., Önal İ.K., Kılıç Z.M.Y., Akdoğan M., Kayaçetin E. (2016). Double balloon enteroscopy: A 7-year experience at a tertiary care centre. Eur. J. Intern. Med..

[18] Di Caro S., May A., Dimitri G., Fini L., Landi B., Petruzziello L., Cellier C., Mulder C., Costamagna G., Ell C. (2005). The European experience with double-balloon enteroscopy: Indications, methodology, safety, and clinical impact. Gastrointest. Endosc..

[19] Moschler O., May A., Muller M.K., Ell C. (2011). Complications in and performance of double-balloon enteroscopy (DBE): Results from a large prospective DBE database in Germany. Endoscopy.

[20] Sheba E., Farag A., Aref W., Elkholy S., Ashoush O. (2017). Double-balloon enteroscopy (DBE) in patients presenting with obscure gastrointestinal bleeding (OGIB). Arab. J. Gastroenterol..

[21] Baek D.H., Hwang S., Eun C.S., Jeon S.R., Kim J., Kim E.R., Yang D.-H., Jang H.J., Im J.P., Park S.J. (2021). Factors Affecting Route Selection of Balloon-Assisted Enteroscopy in Patients with Obscure Gastrointestinal Bleeding: A KASID Multicenter Study. Diagnostics.

[22] Byeon J.-S., Mann N.K., Jamil L.H., Lo S.K. (2012). Double balloon enteroscopy can be safely done in elderly patients with significant comorbidities. J. Gastroenterol. Hepatol..

[23] Wang L., Xie M., Hong L., Zhang C., Zhang T., Fan R., Zhong J., Wang Z. (2020). The diagnostic yields and safety of double-balloon enteroscopy in obscure gastrointestinal bleeding and incomplete small bowel obstruction: Comparison between the adults and elderly. Gastroenterol. Res. Pract..

[24] Zammit S.C., Sanders D., Sidhu R. (2019). Device assisted enteroscopy in the elderly—A systematic review and meta-analysis. Dig. Liver Dis..

[25] Elli L., Scaramella L., Tontini G.E., Topa M., Conte D., Sidhu R., Rondonotti E., Penagini R., Vecchi M. (2022). Clinical impact of videocapsule and double balloon enteroscopy on small bowel bleeding: Results from a large monocentric cohort in the last 19 years. Dig. Liver Dis..

[26] Sidhu R., Sanders D.S. (2013). Double-balloon enteroscopy in the elderly with obscure gastrointestinal bleeding: Safety and feasibility. Eur. J. Gastroenterol. Hepatol..

[27] Choi D.H., Jeon S.R., Kim J.O., Kim H.G., Lee T.H., Lee W.C., Kang B.S., Cho J.-H., Jung Y., Kim W.J. (2014). Double-balloon enteroscopy in elderly patients: Is it safe and useful?. Intest. Res..

[28] Rondonotti E., Spada C., Adler S., May A., Despott E.J., Koulaouzidis A., Panter S., Domagk D., Fernandez-Urien I., Rahmi G. (2018). Small-bowel capsule endoscopy and device-assisted enteroscopy for diagnosis and treatment of small-bowel disorders: European Society of Gastrointestinal Endoscopy (ESGE) Technical Review. Endoscopy.

[29] May A., Nachbar L., Schneider M., Neumann M., Ell C. (2005). Push-and-pull enteroscopy using the double-balloon technique: Method of assessing depth of insertion and training of the enteroscopy. Endoscopy.

[30] Raju G.S., Gerson L., Das A., Lewis B. (2007). American Gastroenterological Association (AGA) Institute medical position statement on obscure gastrointestinal bleeding. Gastroenterology.

[31] World Health Organisation (2010). Definition of an Older or Elderly Person.

[32] ISTAT. http://dati.istat.it/Index.aspx?QueryId=42869.

[33] Johnston C.A., Yung D.E., Joshi A., Plevris J.N., Koulaouzidis A. (2017). Small bowel malignancy in patients undergoing capsule endoscopy at a tertiary care academic center: Case series and review of the literature. Endosc. Int. Open.

[34] Pérez-Cuadrado-Robles E., Zamora-Nava L.E., Jiménez-García V.A., Pérez-Cuadrado-Martínez E. (2018). Indications for and diagnostic yield of capsule endoscopy in the elderly. Rev. Gastroenterol. Mex..

[35] Li L., Chen C., Li Y., Zhang B. (2016). The role of capsule endoscopy in the diagnosis and treatment of obscure gastrointestinal bleeding in older individuals. Eur. J. Gastroenterol. Hepatol..

[36] García-Correa J.J.E., Ramírez-García J.J., García-Contreras L.F., Fuentes-Orozco C., Irusteta-Jiménez L., Michel-Espinoza L.R., Uribe C., Chávez T., González-Ojeda A. (2018). Double-balloon enteroscopy: Indications, approaches, diagnostic and therapeutic yield, and safety. Early experience at a single center. Rev. Gastroenterol. Mex..

[37] Ribeiro Gomes A.C., Pinho R., Rodrigues A., Ponte A., Carvalho J. (2020). Enteroscopy in the Elderly: Review of Procedural Aspects, Indications, Yield, and Safety. GE Port. J. Gastroenterol..

